# Development of an interactive elective “altered anatomy” for students as part of the Z-curriculum according to the NKLM 2.0

**DOI:** 10.3205/zma001625

**Published:** 2023-06-15

**Authors:** Kai Koch, Bernhardt Hirt, Thomas Shiozawa-Bayer, Alfred Königsrainer, Stefano Fusco, Dörte Wichmann

**Affiliations:** 1 University Hospital Tuebingen, Clinic for General, Visceral and Transplant Surgery, Experimental Endoscopy, Research and Development, Tuebingen, Germany; 2 University Hospital Tuebingen, Clinic for General, Visceral and Transplant Surgery, Tuebingen, Germany; 3 University Hospital Tuebingen, Department of Anatomy, Institute for Clinical Anatomy and Cellanalytics, Tuebingen, Germany; 4 University Hospital Tuebingen, Internal Medicine I - Gastroenterology, Gastrointestinal Oncology, Hepatology, Infectiology and Geriatric Medicine, Tuebingen, Germany

**Keywords:** Z-Curriculum, NKLM, modified anatomy

## Abstract

**Objective:**

Many patients have undergone visceral surgery. The effects on anatomy and physiology, which can result in further surgical or gastroenterological clinical pictures, are equally significant and require special knowledge. This content should be taught in an interdisciplinary elective course. The draft of the new 2025 approval regulation and the current approval regulation specify that preclinical and clinical content should specifically be combined within the framework of a Z-curriculum and that the new elective course should meet these requirements.

**Methodology:**

Practical and theoretical aspects of recognising and treating patients with postoperative modified anatomy are to be taught and the findings are to be demonstrated using anatomical and artificial preparations. The curriculum of the preclinical course covers anatomy and physiology. The target group of the curriculum is all participating students with a special interest in topics such as anatomy, visceral surgery and gastroenterology. However, the goal is to involve student tutors of the anatomical dissection courses, who, in turn, will pass on knowledge of modified anatomy to the supervised preclinical students.

**Results:**

According to Thomas and Kern, the curriculum development process entails the following six stages: general needs assessment, targeted needs assessment, the formulation of goals and content, the description of strategies, planned implementation and evaluation.

**Conclusion:**

A “modified anatomy” curriculum for an interdisciplinary elective course in surgery, gastroenterology, and anatomy was developed. Through the training of anatomy table tutors, a “dovetailing” with the preclinical stage is to be achieved. In addition, new concepts related to the transfer of knowledge and competencies were introduced and should be evaluated for suitability.

## Zusammenfassung

**Zielsetzung:**

Viele Patient*innen sind viszeralchirurgisch voroperiert. Die Auswirkungen auf die Anatomie und Physiologie, die weitere chirurgische oder gastroenterologische Krankheitsbilder bedingen können, sind gleichermaßen bedeutsam und benötigen besonderer Kenntnisse. Diese Inhalte sollen in einem interdisziplinären Wahlfach vermittelt werden. Der Entwurf der neuen Approbationsordnung 2025 sowie die geltende Approbationsordnung geben vor, dass gezielt vorklinische und klinische Inhalte im Rahmen eines Z-Curriculums miteinander verbunden werden und das neue Wahlfach diesen Anforderungen gerecht werden sollen.

**Methodik:**

Praktische und theoretische Aspekte im Erkennen und Behandeln von Patient*innen mit postoperativ veränderter Anatomie sollen vermittelt werden und Befunde durch anatomische und artifizielle Präparate demonstriert werden. Inhalte der Vorklinik, die in diesem Curriculum aufgegriffen werden, sind Anatomie und Physiologie. Die Zielgruppe des Curriculums bilden alle teilnehmenden Studierenden, die ein besonderes Interesse an Themen wie Anatomie, Viszeralchirurgie, oder Gastroenterologie haben. Fokussiert sollen jedoch studierende Tutor*Innen der anatomischen Präparier-Kurse eingebunden werden. Durch die Tutor*innen können die Kenntnisse in der veränderten Anatomie an die betreuten Studierenden der Vorklinik weitergegeben werden.

**Ergebnisse:**

Das sechs-schrittige Verfahren zur Curriculums-Entwicklung nach Thomas und Kern wird mit folgenden Etappen beschrieben: allgemeine Bedarfsanalyse, gezielte Bedarfsanalyse, Formulierung von Zielen und Inhalten, Beschreibung der Strategien, geplante Implementierung und Evaluation.

**Schlussfolgerung:**

Ein Curriculum „veränderte Anatomie“ für ein interdisziplinäres Wahlfach in den Fächern Chirurgie, Gastroenterologie und Anatomie wurde entwickelt. Durch die Ausbildung von Anatomie-Tischtutor*Innen soll eine „Verzahnung“ mit der Vorklinik erreicht werden. Neuartige Konzepte der Wissens- und Kompetenzvermittlung wurden eingepflegt und sollten bzgl. Ihrer Eignung evaluiert werden.

## Introduction

Resections and anastomoses can alter the physiological processes of the gastrointestinal tract [1], [2]. Postoperative physicians should understand this. The postoperative modified anatomy results in its own clinical pictures, the knowledge of which, their diagnostics and their therapy are highly relevant for clinical routines [1], [2]. In a newly created curriculum consisting of internal medicine, surgery, and anatomy, the postoperative changes and their effects, according to the Nationaler Kompetenzbasierter Lernzielkatalog Medizin (NKLM), are to be specifically taught and linked in an interdisciplinary approach. The curriculum is offered as an elective course in “postoperative modified anatomy”. It considers the draft of the current “National Competency-Based Learning Objectives Catalogue Medicine 2.0 (NKLM)” and was designed according to Thomas et al. “Curriculum Development for Medical Education: A Six-Step Approach” [3], [https://nklm.de/zend/menu]. 

In the following, the development of the “postoperative modified anatomy” curriculum is presented based on the six-step approach of Thomas et al.

## Step 1: General needs assessment

The number of cases of resective and reconstructive surgery of the upper and lower gastrointestinal tract have been increasing in recent years [4], [5]. With a growing number of patients in daily, clinical care, modified anatomy occurs as a result of this development. Therefore, knowledge and understanding of postoperative modified digestive physiology and possible pathophysiological processes are of increasing importance for epidemiological reasons [1], [6].

With the claim made by “Masterplan Medical Studies 2020” that “[...] the consistent orientation towards patients and their needs should be learnt and practised at an early stage [...]” and “[...] the next generation of physicians should be prepared as well as possible for the requirements in the medical profession [...]”, another challenge for the curriculum development of teaching and clinically active physicians arises [7]. Therefore, the care of patients with postoperative modified anatomy should not only be learnt and addressed in the context of medical practice. A sufficient basis for later patient care should already be developed in the course of studies by teaching the relevant content.

Structurally, the implementation of the draft of the new medical licensing regulations (ÄApproO) from 2025 will result in the need to link preclinical and clinical content more closely [8]. The National Competence-Based Learning Objectives Catalogue Medicine specifies which content is relevant for students of human medicine “[...] nationwide [...]” [9]. Which competencies are to be learnt in detail by the students and, in the best case, understood, is thus predetermined. In the draft for the new ÄApprO from 2025, the recommendation letter from the expert commission for the implementation of the “Masterplan Medical Studies 2020” to provide for medical competencies no longer requires the strict separation of clinical and preclinical content [https://nklm.de/zend/menu], [7], [10]. The term “Z-curriculum” from the Masterplan 2020 stands for the “identification” of preclinical knowledge and clinical aspects [7]. Based on the development of an elective course in “postoperative modified anatomy”, this interlocking is to be made possible by students of the clinical semesters working as table tutors in anatomy

According to the specifications of the new NKLM, learning content is understood and treated on several levels. NKLM 2.0 provides for four different “depths of competence”, comprising cognition (1), affection (2), and action competence (3A & 3B) [https://nklm.de/zend/menu]. The challenge for curriculum development is to design a meaningful combination of the content to be taught and the corresponding depth of competence. Various teaching strategies are available to teach individual competencies [3]. In addition, the Z-curriculum calls for a basic in-depth study (“G”) of preclinical content within the framework of clinical training [https://nklm.de/zend/menu].

## Step 2: Targeted needs analysis

As already mentioned, according to the “Masterplan Medical Studies 2020”, “[...] the next generation of physicians should be prepared as well as possible for the requirements in the medical profession [...]” [7]. Furthermore, the medical licensing regulations stipulate that “[...] the basic knowledge about bodily functions and the mental-emotional characteristics of humans [...]” and “[...] the basic knowledge about diseases and sick humans [...]” should form the content of medical studies [https://www.gesetze-im-internet.de/_appro_2002/BJNR240500002.html]. At the same time, neither the additional training regulations for specialists in visceral surgery nor the additional training regulations for specialists in gastroenterology specifically include interdisciplinary content on modified anatomy [11]. This results in the problem of adequate care for patients with altered anatomy who must be treated based on internal medicine or surgery-related clinical pictures [12]. 

Therefore, sufficient training in the basics of visceral medical clinical pictures is also relevant to the education of students. The present draft of the NKLM forms a suitable guideline: “The new learning objectives catalogue of NKLM defines competencies that are based on the professional profile of the physician and the dentist and that should be available after the completion of the respective studies [...]” [https://nklm.de/zend/menu]. Moreover, as shown in table 1 (Tab. 1), it already contains much interdisciplinary content that anticipates further specialist training.

A well-integrated curriculum should be tailored to the environment in which it is used. In this case, a curriculum was intentionally developed that intersects different specialities across different study sections. Clinical contents such as surgery and internal medicine, specifically visceral surgery and gastroenterology, incorporate preclinical content in anatomy and physiology. In the future, particularly due to the new “NKLM 2.0”, the preclinical basics for clinical students could also become more prominent in the clinical section.

In the planned curriculum, clinical anatomy table tutors should also be trained to recognise altered body donor anatomies and to understand anatomy’s implications. To date, there are no described teaching opportunities for table tutors to recognise and describe postoperative modified anatomy of body donors. It should be noted that most table tutors are clinical students. Thus, the table tutors can teach the required preclinical content and valuable clinical content can also be taught through the knowledge gained from the elective course “postoperative modified anatomy”. 

The effectiveness of similar forms of peer-to-peer teaching has already been demonstrated in various scenarios [13], [14], [15]. They should be able to independently pass on the knowledge to preclinical students and, thus, benefit from the “peer-to-peer” effect themselves [15].

## Step 3: Goals and content

Four subject areas in the NKLM were identified, hereafter referred to as learning objectives (A–D), which are addressed in the curriculum. The learning objectives and the competencies to be taught are listed in table 1 (Tab. 1). For learning objective C, there is no assignment in the NKLM.

Both Thomas et al. and the NKLM assign the requirements to so-called taxonomies. These taxonomies represent depths of competence to be developed by the curriculum. In the NKLM, the depths of competence are divided into knowledge (W) and action (H) and assigned to the numbers 1-3 [3], [https://nklm.de/zend/menu]. Table 2 (Tab. 2) explains the term depth of competence with the corresponding descriptor.

## Step 4: Strategies

In this section, the learning objectives mentioned in step 3 are assigned to depths of competence according to the taxonomy to identify the corresponding strategies. Each depth of competence has different strategies to target the content; furthermore, the strategies have advantages and disadvantages [16], [17], [18]. There is no clear, evident proof of the appropriate teaching strategy for an appropriate target because different students may also be different types of learners in each case [19]. Nevertheless, efficient methods can be used for depth of competence. For example, lectures and case studies are appropriate because of their effectiveness and resource efficiency [20], [21]. However, in competency depths 2 and 3, where contexts are explained and understood and actions are to be performed independently, classical teaching strategies such as lectures are considered less suitable [3]. For this reason, innovative and sustainable teaching strategies should be implemented for depth of competence 3. Simulations and training tasks can achieve sustainable effects in these depths of competence [22], [23]. Table 3 (Tab. 3) breaks down the specific requirements from step 2 as required learning objectives. The verbs describing the learning objectives are assigned to the competency levels (1-3) described by the NKLM according to their taxonomy (example, verb: “describe”, taxonomy: knowledge, competency level: 1). Which competencies are required is defined by the choice of the learning objective. The verbs that describe the learning objective are decisive in each case.

**Figure 1:**
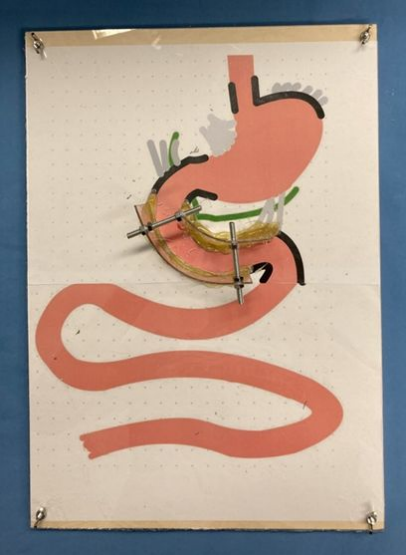
Interactive “Altered anatomy Simulators”: A printed image of the anatomy is glued onto a wooden plate. The parts of the anatomy that are circled in black represent boundaries that should not be mobilized in real patients (e.g. the retroperitoneum). Shown in gray are the stabilizing ligaments that could be mobilized during various maneuvers. Along the black line, acrylic glass boundaries are glued on both sides so that students cannot move the plasticine on the plate. The dough can only be mobilized and moved in the free areas. The anatomy template is also sealed with an acrylic glass plate and screwed onto the wooden mold. The materials used can all be purchased inexpensively at a hardware store.

For the depths of competence 3a & 3b, which are particularly required in the present project, training materials were created independently (printed in bold in table 3 (Tab. 3), see figure 1 (Fig. 1)). These are designed to sustainably teach independent (and guided) action skills and, thus, are different from the rest of the teaching strategies and competency depths 1W and 2W (explained in table 2 (Tab. 2)).

Interactive learning materials are used to innovatively teach modified anatomy, specifically Roux-Y anatomy. For this purpose, “altered anatomy simulators” were created. They consist of a wooden board to which a laminated poster is glued. Using this poster as a base, students are asked to recreate regular anatomy with modelling clay. The idea was based on a teaching video by Lars Aabakken on “postoperative modified anatomy” [24]. In the places where the anatomy is not very moveable in the real patient due to special structures (for example, Treitz's ligament), there are limitations on the template. In these places, the plasticine cannot be mobilised. Students should specifically separate the areas of the plasticine organs that are easy to mobilise and “anastomose” them to each other. This provides a deeper understanding of the anatomy. The templates should be inexpensive to create based on the setup in figure 1 (Fig. 1) and easy to replicate by other faculty members.

The timeline for implementing the elective is shown in table 4 (Tab. 4). 

## Step 5: Implementation

The planned curriculum for an elective is to be offered to clinical students in the 5^th^ to 10^th^ semester. According to the ÄAppO, an elective subject should be completed in the clinical study section, which is not understood to be a uniform offering [https://www.gesetze-im-internet.de/_appro_2002/BJNR240500002.html]. At the Medical Faculty of the University Hospital of Tübingen, electives can be completed in the areas of the “research-oriented Tübingen clinical curricula” (TüKliF) or the “clinical curricula specials” (TüKliS), with a minimum of 40 h [25]. The present curriculum is to take place during a 10-hour elective course in “TüKliS” in surgery. Students can register for the course via the existing and established online portal of the Faculty of Medicine.

Planning the implementation includes the analysis of the necessary resources, the available resources, and the resources to be acquired [3]. In addition to organisational elements, the main components of resources are learning materials and personnel. The learning materials for the above-mentioned contents are partly available from the existing anatomy, surgery, and internal medicine curricula and are compiled by the teaching staff. They are intended to specifically reflect the curriculum described. The lecturers are physicians from the Departments of General, Visceral and Transplant Surgery, the Medical Clinic, Internal Medicine I, and the Institute of Clinical Anatomy at the University Hospital of Tübingen. This involves lectures, case studies, and demonstration videos. Separately considered are the newly created learning materials (see figure 1 (Fig. 1)), which were designed according to the requirements by the depths of competence 3a and 3b and are newly created for the curriculum.

## Step 6: Evaluation

Primarily, three user groups are available for the planned evaluation:

- Participants
- Faculty
- Curriculum organisation

All three user groups benefit from feedback and have their own interests. For participants, the focus is on gains in content-related knowledge and their own performance. Faculty and curriculum organisations benefit from feedback on teaching strategies, content, and organisation. This knowledge can be used for future interdisciplinary curricula. For the curriculum organisation, points such as the elective structure, concrete implementation, and the handling of the simulators created are especially important. The result of this breakdown is that students should be evaluated in the interest of all user groups, including the faculty and the organisation. The following evaluation design is appropriate for the planned elective: 

*Pretest* during the first hour of the elective, post-test at the end of the elective, and subsequent comparison of learning outcomes.

The content of the exam consists mainly of multiple-choice questions to test the depths of competency 1 and 2, but also includes a separate task. To approximate and inquire about the depth of competence 3, students are asked to draw different anatomies before and after resection in both tests. In this way, the learning effect for depth of competency 3a can be assessed at the completion of the elective. Also planned is a programmatic evaluation of organisation and teaching strategies. The evaluation design would allow statistical tests to measure the significance of possible differences between evaluation scores. The evaluation data collected would be used to examine and adjust teaching strategies, general course content, and specific course content. Regular evaluation of the elective should result in the curriculum being optimised with each cycle.

## Summary

The planning of an interdisciplinary elective course ‘Postoperative Modified Anatomy’ by the Department of General, Visceral and Transplant Surgery, the Medical Clinic I with Gastroenterology and the Institute of Clinical Anatomy is presented. It has been possible to develop an interlinked curriculum in six steps which benefits clinical and preclinical students. The individually required depths of competence of the NKLM 2.0 can be learnt based on the present curriculum with the respective relevant strategies. At the same time, in the sense of the Z-curriculum, clinical students can repeat the newly required “basic knowledge” in the clinical section and deepen it using clinical examples. The implementation of the curriculum takes place on a tightly timed schedule. The constantly growing knowledge of future physicians makes the efficient design of medical courses urgently necessary. Through evaluation, the developed curriculum contains a mechanism through which findings on the suitability of teaching methods, teaching content, and the organisation can be further developed and considered in repeated implementations. The evaluations will show whether a Z-curriculum in the sense of the new licensing regulations can be implemented based on the elective subject “postoperative modified anatomy”. A blueprint for the further planning of courses according to the specifications of a Z-curriculum is presented to the reader here. 

## Funding

At the Faculty of Medicine of the University Hospital Tübingen, the funding line PROFIL is awarded by the Quality Offensive Teaching, which made the present work possible (F.7231105).

## Competing interests

The authors declare that they have no competing interests. 

## Figures and Tables

**Table 1:**
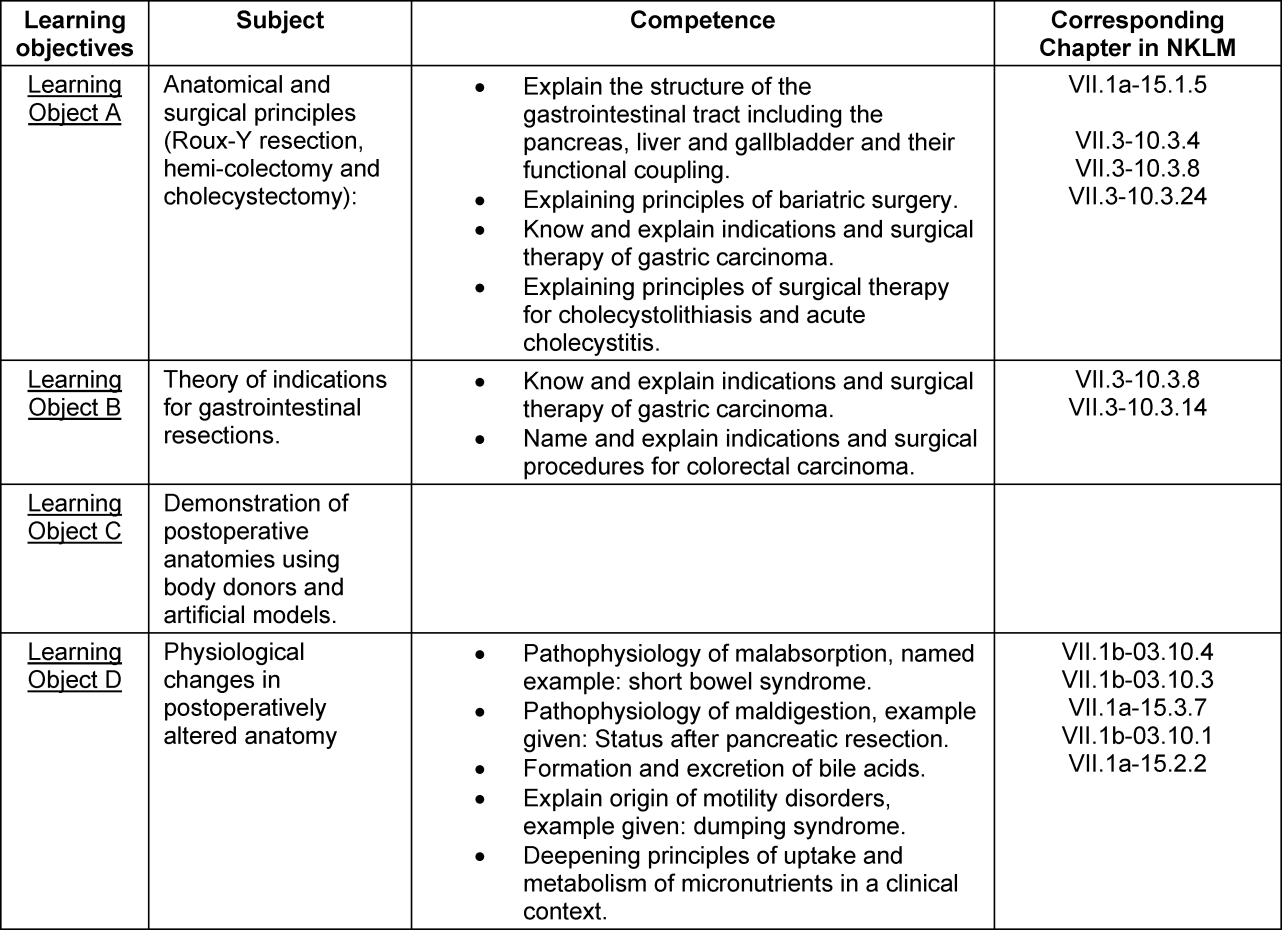
The learning objectives are summarized in terms of subjects. For each subject, the appropriate competencies from the new NKLM 2.0 can be found and cited (4), page 5

**Table 2:**
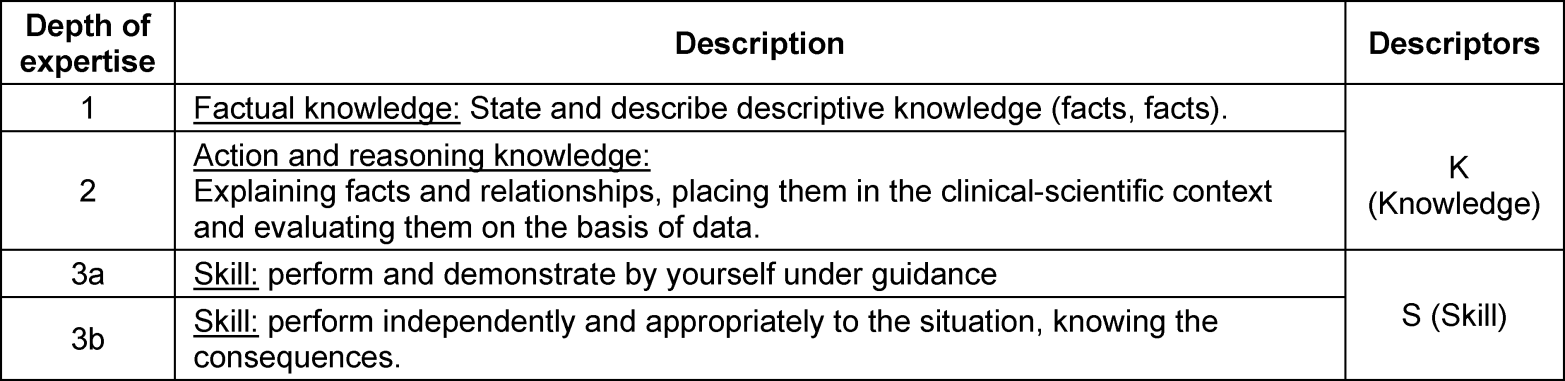
Abstract of the LOOP Learning Objectives Catalog, description of the different depths of competencies 1 -3b (4), page 5

**Table 3:**
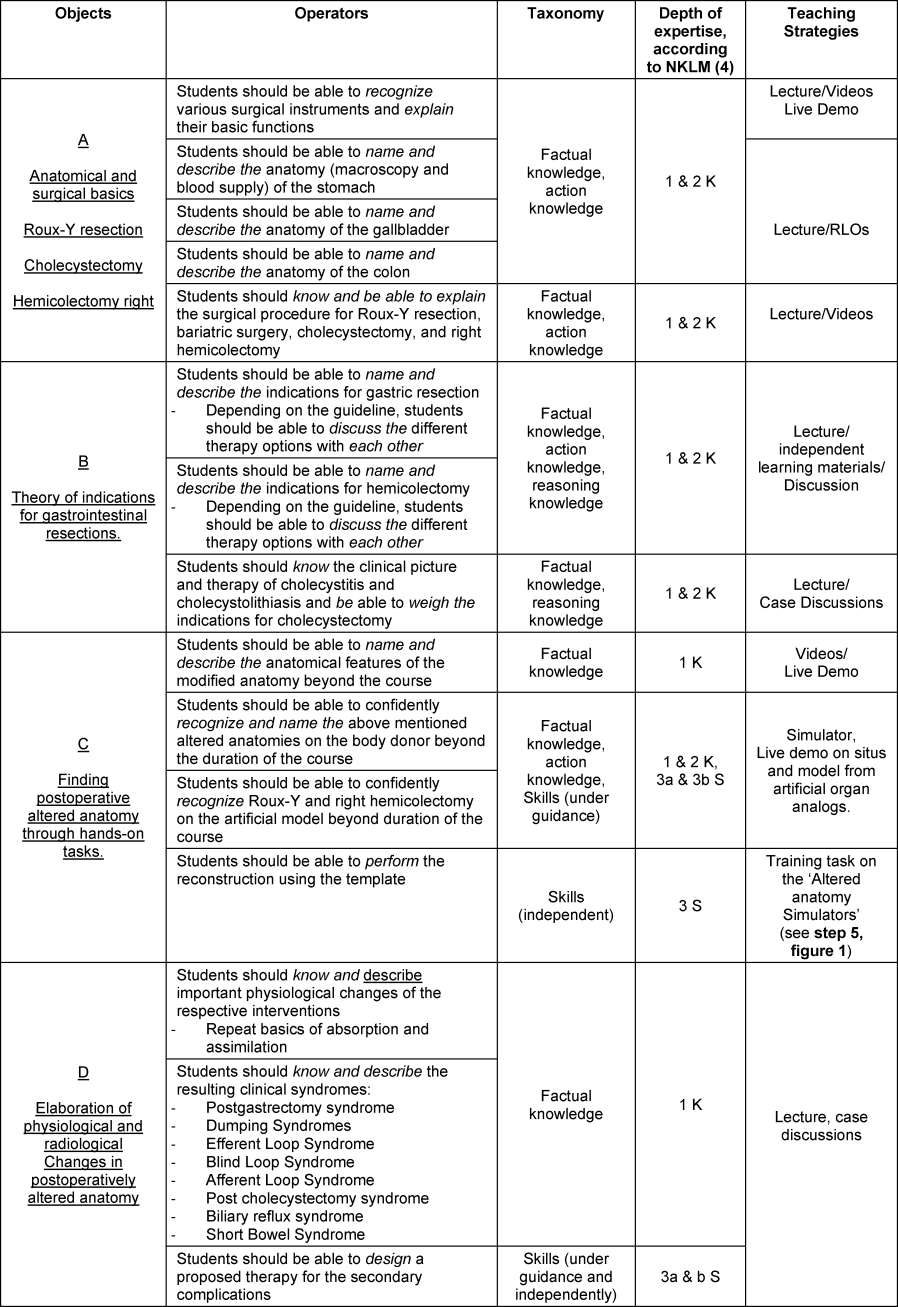
Learning objectives (A-D) with assigned concrete skills, corresponding taxonomy and teaching strategies, page 5

**Table 4:**
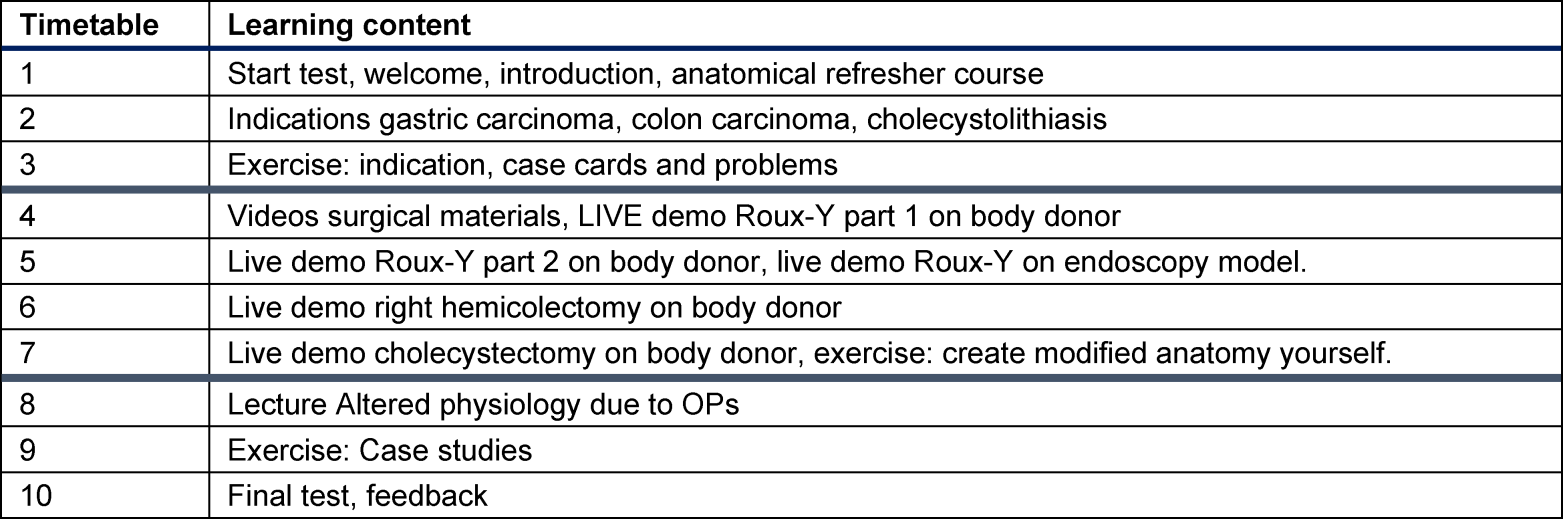
Schedule of the elective

## References

[1] Davis JL, Ripley RT (2017). Postgastrectomy Syndromes and Nutritional Considerations Following Gastric Surgery. Surg Clin North Am.

[2] Massironi S, Cavalcoli F, Rausa E, Invernizzi P, Braga M, Vecchi M (2020). Understanding short bowel syndrome: Current status and future perspectives. Dig Liver Dis.

[3] Thomas PA, Kern DE, Hughes MT, Chen BY (2016). Curriculum Development for Medical Education: A Six-Step Approach.

[4] Statistisches Bundesamt Entgeltsysteme im Krankenhaus, DRG-Statistik und PEPP-Statistik "Operationen und Prozeduren der vollstationären Patientinnen und Patienten in Krankenhäusern 2005-2018".

[5] English WJ, DeMaria EJ, Brethauer SA, Mattar SG, Rosenthal RJ, Morton JM (2018). American Society for Metabolic and Bariatric Surgery estimation of metabolic and bariatric procedures performed in the United States in 2016. Surg Obes Relat Dis.

[6] Welbourn R, Hollyman M, Kinsman R, Dixon J, Liem R, Ottosson J, Ramos A, Våge V, Al-Sabah S, Brown W, Cohen R, Walton P, Himpens J (2019). Bariatric Surgery Worldwide: Baseline Demographic Description and One-Year Outcomes from the Fourth IFSO Global Registry Report 2018. Obes Surg.

[7] Bundesministerium für Bildung und Forschung (2017). Masterplan Medizinstudium 2020.

[8] Bundesministerium für Gesundheit Verordnung zur Neuregelung der ärztlichen Ausbildung. Referentenentwurf des Bundesministeriums für Gesundheit.

[9] Medizinischer Fakultätentag (2015). Nationaler Kompetenzbasierter Lernzielkatalog Medizin (NKLM).

[10] Wissenschaftsrat (2018). Neustrukturierung des Medizinstudiums und Änderung der Approbationsordnung für Ärzte. Empfehlungen der Expertenkommission zum Masterplan Medizinstudium 2020. Drs. 7271-18.

[11] Landesärztekammer Baden-Württemberg (2020). Neufassung der Weiterbildungsordnung der Landesärztekammer Baden-Württemberg, vom 18. Mai 2020.

[12] Moreels TG (2013). ERCP in the patient with surgically altered anatomy. Curr Gastroenterol Rep.

[13] Zhu A, Deng S, Greene B, Tsang M, Palter VN, Jayaraman S (2021). Helping the Surgeon Recover: Peer-to-Peer Coaching after Bile Duct Injury. J Am Coll Surg.

[14] Chan E, Doroudgar S, Huang J, Ip EJ (2020). Interprofessional Education on Medication Adherence: Peer-to-Peer Teaching of Osteopathic Medical Students. J Am Osteopath Assoc.

[15] Benè KL, Bergus G (2014). When learners become teachers: a review of peer teaching in medical student education. Fam Med.

[16] Breytenbach C, Ten Ham-Baloyi W, Jordan PJ (2017). An Integrative Literature Review of Evidence-Based Teaching Strategies for Nurse Educators. Nurs Educ Perspect.

[17] Wang P, Ma T, Liu LB, Shang C, An P, Xue YX (2021). A Comparison of the Effectiveness of Online Instructional Strategies Optimized With Smart Interactive Tools Versus Traditional Teaching for Postgraduate Students. Front Psychol.

[18] Berg RM, Plovsing RR, Damgaard M (2012). Teaching baroreflex physiology to medical students: a comparison of quiz-based and conventional teaching strategies in a laboratory exercise. Adv Physiol Educ.

[19] Rohrer D, Pashler H (2012). Learning styles: where’s the evidence?. Med Educ.

[20] Thistlethwaite JE, Davies D, Ekeocha S, Kidd JM, MacDougall C, Matthews P, Purkis J, Clay D (2012). The effectiveness of case-based learning in health professional education. A BEME systematic review: BEME Guide No. 23. Med Teach.

[21] Bourne PE (2007). Ten Simple Rules for Making Good Oral Presentations. PLOS Comput Biol.

[22] Issenberg SB, Petrusa ER, McGaghie WC, Felner JM, Waugh RA, Nash IS, Hart IR (1999). Effectiveness of a computer-based system to teach bedside cardiology. Acad Med.

[23] Zhang W, Liu X, Zheng B (2021). Virtual reality simulation in training endoscopic skills: A systematic review. Laparosc Endosc Robot Surg.

[24] Endoscopy Campus, Lars Aabakken Lars explains Anatomy-Y Roux Anatomie nach Magenresektion.

[25] Dekanat der Medizinischen Fakultät Tübingen (2019). Medizinische Fakultät Bereich Studium und Lehre, Leitfaden Studiengang Medizin.

